# The Role of Butyric Acid in Treatment Response in Drug-Naïve First Episode Schizophrenia

**DOI:** 10.3389/fpsyt.2021.724664

**Published:** 2021-08-23

**Authors:** Xue Li, Xiaoduo Fan, Xiuxia Yuan, Lijuan Pang, Shaohua Hu, Yunpeng Wang, Xufeng Huang, Xueqin Song

**Affiliations:** ^1^Department of Psychiatry, The First Affiliated Hospital of Zhengzhou University, Zhengzhou, China; ^2^Biological Psychiatry International Joint Laboratory of Henan, Zhengzhou University, Zhengzhou, China; ^3^Henan Psychiatric Transformation Research Key Laboratory, Zhengzhou University, Zhengzhou, China; ^4^Psychotic Disorders Program, UMass Memorial Medical Center, University of Massachusetts Medical School, Worcester, MA, United States; ^5^Center for Neuroscience and Department of Psychiatry of First Affiliated Hospital, Zhejiang University School of Medicine, Hangzhou, China; ^6^The Key Laboratory of Mental Disorder Management in Zhejiang Province, Brain Research Institute of Zhejiang University, Hangzhou, China; ^7^Centre for Lifespan Changes in Brain and Cognition (LCBC), Department of Psychology, University of Oslo, Oslo, Norway; ^8^Illawarra Health and Medical Research Institute, University of Wollongong, Wollongong, NSW, Australia

**Keywords:** schizophrenia, butyric acid, treatment response, risperidone, GC-MS

## Abstract

**Background:** Butyric acid, a major short-chain fatty acid (SCFA), has an important role in the microbiota–gut–brain axis and brain function. This study investigated the role of butyric acid in treatment response in drug-naïve first episode schizophrenia.

**Methods:** The study recruited 56 Chinese Han schizophrenia inpatients with normal body weight and 35 healthy controls. Serum levels of butyric acid were measured using Gas Chromatography-Mass Spectrometer (GC-MS) analysis at baseline (for all participants) and 24 weeks after risperidone treatment (for patients). Clinical symptoms were measured using the Positive and Negative Syndrome Scale (PANSS) for patients at both time points.

**Results:** At baseline, there was no significant difference in serum levels of butyric acid between patients and healthy controls (*p* = 0.206). However, there was a significant increase in serum levels of butyric acid in schizophrenia patients after 24-week risperidone treatment (*p* = 0.030). The PANSS total and subscale scores were decreased significantly after 24-week risperidone treatment (*p's* < 0.001). There were positive associations between baseline serum levels of butyric acid and the reduction ratio of the PANSS total and subscale scores after controlling for age, sex, education, and duration of illness (*p's* < 0.05). Further, there was a positive association between the increase in serum levels of butyric acid and the reduction of the PANSS positive symptoms subscale scores (*r* = 0.38, *p* = 0.019) after controlling for potential confounding factors.

**Conclusions:** Increased serum levels of butyric acid might be associated with a favorable treatment response in drug-naïve, first episode schizophrenia. The clinical implications of our findings were discussed.

## Introduction

Schizophrenia is a chronic, severe, and disabling neuropsychiatric disorder that affects ~1% of the world population and imposes an enormous burden on society (1, 2). The underlying mechanism of schizophrenia pathogenesis has yet to be fully understood. In recent years, the microbiota-gut-brain axis has arisen as a major topic of interest in psychiatry. Dysregulation in the composition of the gut microbiota has been identified in schizophrenia (3). Emerging clinical and preclinical studies suggest a role for the gut microbiota in schizophrenia (4, 5).

Short-chain fatty acids (SCFAs), the main metabolites produced by gut microbiota, are believed to play a key role in microbiota–gut–brain crosstalk (6). SCFAs are defined as 1–6 carbon fatty acids that have a straight or branched conformation (7). Acetate, propionate, and butyrate are the three most common SCFAs and together constitute 85–89% of the SCFAs in human serum (8). Accumulating data in the literature strongly indicate that butyrate can cross the blood-brain barrier and its concentrations in wet brain samples are about an order of magnitude higher than those reported in peripheral blood (9, 10). Studies have suggested that butyrate may have a beneficial role in several neuropsychiatric conditions such as Alzheimer disease, Parkinson disease, autism, and schizophrenia (11–13). Butyrate is able to protect neurons from cell death in animal models of Parkinson disease by reversing the disease-associated reduction in histone acetylation (14, 15). *In vivo* study found that patients with schizophrenia have histone deacetylases dysregulation (16). In addition, recent studies have showed that abnormal fecal butyrate levels may play an important role in the pathogenesis of autism spectrum disorder (ASD) (17, 18). The purpose of our study was to examine the role of butyric acid in treatment response in patients with drug-naïve, first episode schizophrenia.

## Methods

### Participants

From July 2017 to December 2018, Chinese Han drug-naïve, first episode schizophrenia patients with normal body weight were recruited from the psychiatric inpatient wards of the First Affiliated Hospital of Zhengzhou University. Healthy controls were recruited from the local community through advertising during the same time period. This study was approved by the Human Ethics Committee of the First Affiliated Hospital of Zhengzhou University, China (Approval No. 2016-LW-17). Written informed consents were obtained from all subjects after complete descriptions provided.

The inclusion criteria for patients were: (1). met the criteria for Diagnostic and Statistical Manual of Mental Disorders fourth version (DSM-IV) schizophrenia; two independent psychiatrists used the Structured Clinical Interview for DSM-IV (SCID) to make the diagnosis (19, 20); (2). never treated with antipsychotics before; (3). born through normal vaginal delivery; (4). normal body weight (BMI: 18.5–23.9); (5). between 18 and 45 years of age. The exclusion criteria for patients included: (1). diagnoses of autoimmune diseases, hepatobiliary and gastrointestinal diseases, blood diseases, diabetes, neurological diseases, or psychiatric diseases other than first-episode schizophrenia; (2). pregnant or lactating women; (3). significant diarrhea or constipation in the past month; (4). a significant change in the living environment or diet in the past month; (5). a history of using any antibiotic or anti-inflammatory agent, or probiotic in the past month. Healthy controls were evaluated by a research psychiatrist to rule out any psychiatric or medical conditions. Healthy controls must fit the same inclusion and exclusion criteria as patients except the schizophrenia diagnosis.

### Clinical Symptoms Assessment

The psychopathology of patients was evaluated using the Positive and Negative Syndrome Scale (PANSS) by two research psychiatrists together. The PANSS is a standardized, semi-structured clinical interview that rates the presence and severity of positive and negative symptoms, as well as general psychopathology for people with schizophrenia within the past week. Of the 30 items, seven are positive symptoms, seven are negative symptoms, and 16 are general psychopathology symptoms. Symptom severity for each item is rated according to which anchoring points in the 7-point scale (1 = absent; 7 = extreme) best describe the presentation of the symptom (21). After baseline assessment, all patients were treated with risperidone in the dose range from 1 mg/day to 4–6 mg/day based on the clinical judgment of treating psychiatrists. No other medication was allowed during the study except benzodiazepines for insomnia and anticholinergic agents for dystonia reaction. The PANSS was administered again to all patients after 24-week risperidone treatment.

### Laboratory Tests

After fasting overnight, venous blood was collected between 7:00 and 8:00 A.M. for all subjects at baseline; the PANSS was administered to patients on the same day. For patients, fasting blood samples were collected and the PANSS was administered on the same day again after 24-week risperidone treatment. Serum was obtained through centrifugation at 3,000 rpm for 10 min, then transferred into Eppendorf tubes, and stored at −70°C for SCFA assay. SCFAs standards were prepared and stored at 0°C for use. In brief, 100 μL serum samples were vortexed in 50 μL of 15% phosphoric acid with 10 μL of 75 μg/mL isohexanoic acid solution as internal standard and 140 μL ether (30 s). Subsequently, the samples were centrifuged at 4°C for 10 min (12,000 rpm) and the supernatant was transferred into the vial prior to Gas Chromatography-Mass Spectrometer (GC-MS) analysis. The GC was fitted with a capillary column Agilent HP-INNOWAX (30 m ^*^ 0.25 mm i.d. ^*^ 0.25 μm) (Agilent Technologies, Santa Clara, CA, USA) and helium was used as the carrier gas at 1 mL/min. Injection was made in split mode at 10:1 with an injection volume of 1 μL and an injector temperature of 250°C. The temperature of the ion source, interface, and quadrupole were 230, 250, and 150°C, respectively. The column temperature was initially 90°C, then increased to 120°C at 10°C/min, to 150°C at 5°C/min, and finally to 250°C at 25°C/min and kept at this temperature for 2 min (total run-time of 15 min). The detector was operated in electron impact ionization mode (electron energy 70 eV) using full scan and selected ion monitoring (SIM) mode. The quantity of each sample was analyzed with a calibration curve using standard chemicals.

### Statistical Analysis

The data were analyzed using SPSS version 25.0 (IBM, Armonk, NY). Normality was tested with the Shapiro-Wilk test, and equal variance was evaluated before analysis of variance analysis. To compare demographic and clinical variables between groups, we used χ^2^ test for categorical variables and Student's *t*-test for continuous variables. The reduction ratio of the PANSS score was defined as (baseline PANSS score minus 24-week PANSS score)/baseline PANSS score. A 50% or greater reduction ratio was defined as “good” response (22). The longitudinal data refer to the population of completers, not to the intent-to-treat population. In order to take into account both non-completers and completers in longitudinal analyses, a linear mixed model approach was used. The PANSS scores and serum levels of butyric acid were used as dependent variables and age, sex, education, duration of illness were chosen as the fixed effect variables. Pearson correlation and partial correlation analyses were performed to examine the relationships between baseline serum levels of butyric acid or changes of butyric acid after 24-week risperidone treatment and the reduction ratios of the PANSS scores. Logistic regression analysis was used to examine the predictive value of serum levels of butyric acid at baseline to treatment response at week 24. Two-tailed significance values were used and significance levels were set at 0.05.

## Results

### Baseline Comparison Between Patients and Healthy Controls

**Figure 1:**
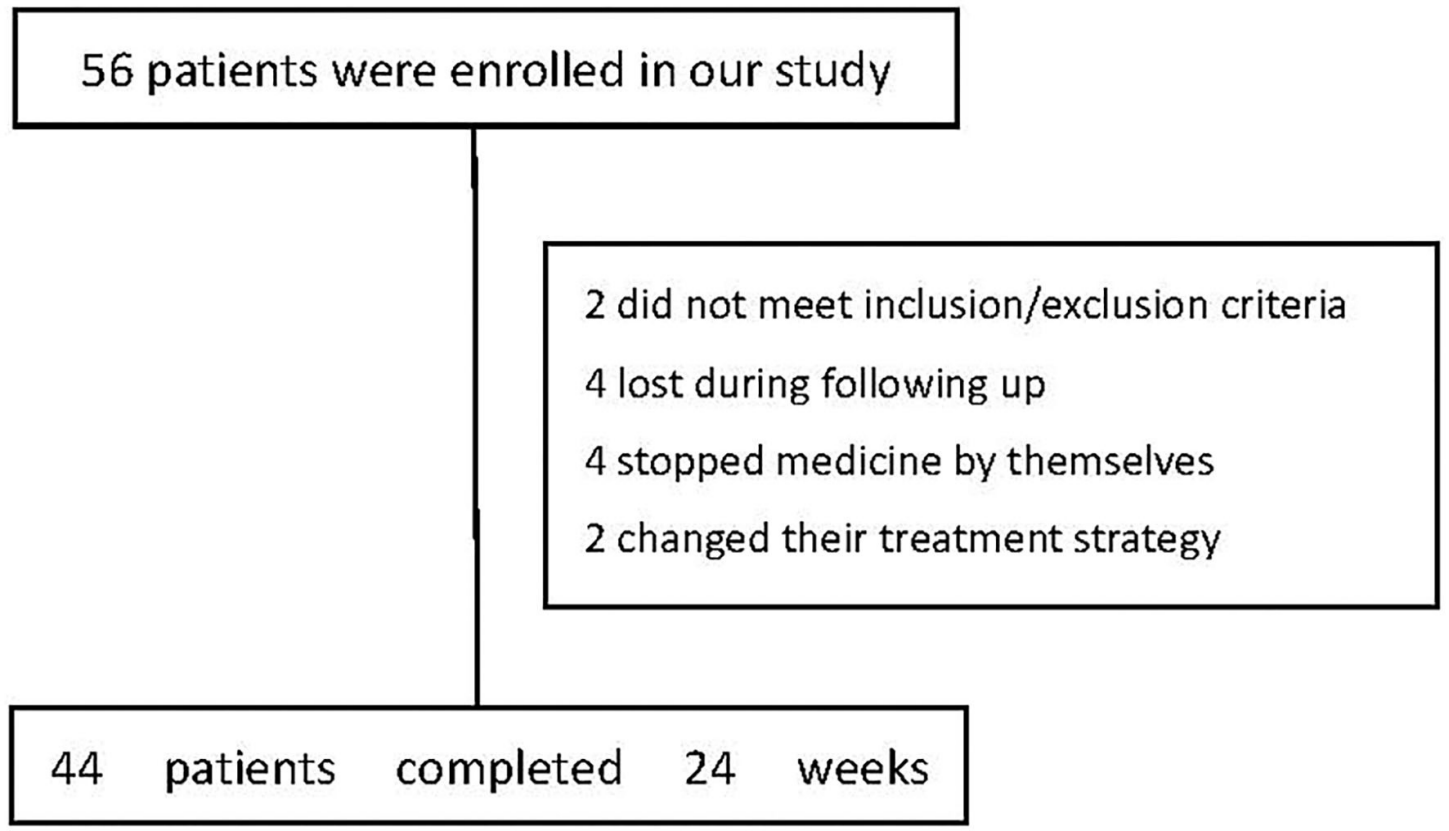
The flow chart of patients at baseline and 24 weeks after treatment.

Fifty-six drug-naïve, first-episode schizophrenia patients were screened and 54 were enrolled because they met the inclusion and exclusion criteria. Forty-four patients completed the 24-week assessment (Figure 1). Thirty-five healthy controls were recruited in the study and completed the baseline assessment only. The baseline characteristics of the subjects were summarized in Table 1. There were no significant differences between the two groups in age, education, sex, marital status, body mass index (*p*'s > 0.05). In addition, there was no significant difference between the two groups in serum levels of butyric acid (*p* = 0.206) (Table 1). No significant difference at baseline in neither demographic nor clinical parameters was observed between the patients that dropped out of follow-ups and those remained until the end of the study (*p*'s > 0.05).

**Table 1:** Demographics and baseline serum levels of butyric acid in schizophrenia patients and healthy controls.

		**Schizophrenia (*n* = 54)**	**Control (*n* = 35)**	**T/χ^**2**^**	***p*-value**
Age (year) (mean ± SEM)		22.6 ± 0.6	23.7 ± 0.5	−1.36	0.180
Education (year) (mean ± SEM)		11.5 ± 0.4	12.3 ± 0.5	−1.40	0.165
Duration of illness (month) (mean ± SEM)		8.6 ± 1.0			
Family history of psychosis, n%		7/54 (12.9%)			
Sex	Male, n%	17 (31.5%)	11 (31.4%)	0.00	0.97
	Female, n%	37 (68.5%)	24 (68.6%)		
Married, n%		9 (16.6%)	9 (25.7%)	1.08	0.299
Body weight (kg) (mean ± SEM)		55.4 ± 1.3	58.6 ± 1.5	−1.61	0.111
Body mass index (kg/m^2^) (mean ± SEM)		20.4 ± 0.3	21.2 ± 0.4	−1.88	0.065
Butyric acid (μg/mL) (mean ± SEM)		0.12 ± 0.01	0.10 ± 0.01	1.63	0.107

### The Changes in the PANSS Scores and Serum Levels of Butyric Acid After 24-Week Risperidone Treatment

We found that the PANSS total, positive symptoms, negative symptoms, and general psychopathology subscale scores significantly decreased after 24-week risperidone treatment (*p*'s <0.001). The serum levels of butyric acid were significantly increased after 24-week risperidone treatment (*p* = 0.030) (Table 2).

**Table 2:** The serum levels of butyric acid and PANSS total and subscale scores in patients at baseline and 24 weeks after treatment.

		**Baseline**	**24 Weeks**	***F***	***p*-value**
Butyric acid (μg/mL) (mean ± SEM)		0.12 ± 0.01	0.14 ± 0.01	−2.24	**0.030**
PANSS	Positive (mean ± SEM)	21.23 ± 0.74	10.18 ± 0.29	29.13	** <0.001**
	Negative (mean ± SEM)	21.57 ± 0.86	12.98 ± 0.43	18.89	** <0.001**
	General (mean ± SEM)	43.09 ± 1.05	27.66 ± 0.91	26.30	** <0.001**
	Total (mean ± SEM)	85.89 ± 2.10	50.82 ± 1.54	31.64	** <0.001**

*The value of the bold means p <0.05*.

**Figure 2:**
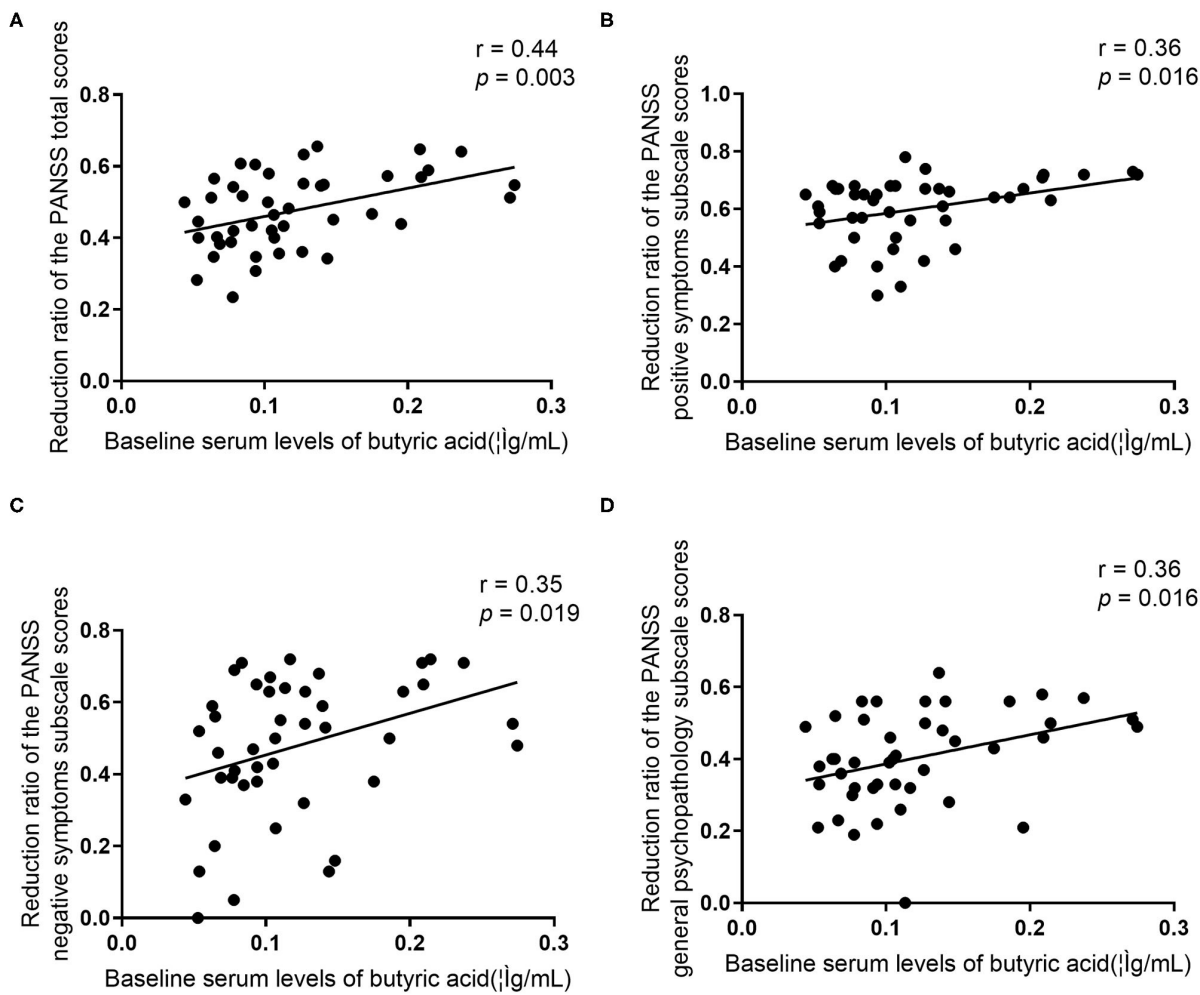
(**A–D**) Relationships between the reduction of the PANSS total scores or subscale scores and the increase in serum levels of butyric acid in patients.

We further found that there was a positive association between the reduction of the PANSS positive symptoms subscale scores and the increase in serum levels of butyric acid after 24-week treatment in schizophrenia patients (*r* = 0.34, *p* = 0.025) (Figure 2). After controlling for possible confounding variables including age, sex, education, and duration of illness, BMI, the correlation remained significant (*r* = 0.38, *p* = 0.019). There were no significant correlations between the reduction of PANSS total scores or other subscale scores and the increase in serum levels of butyric acid (*p's* > 0.05).

### Baseline Serum Levels of Butyric Acid and Treatment Response

**Figure 3:**
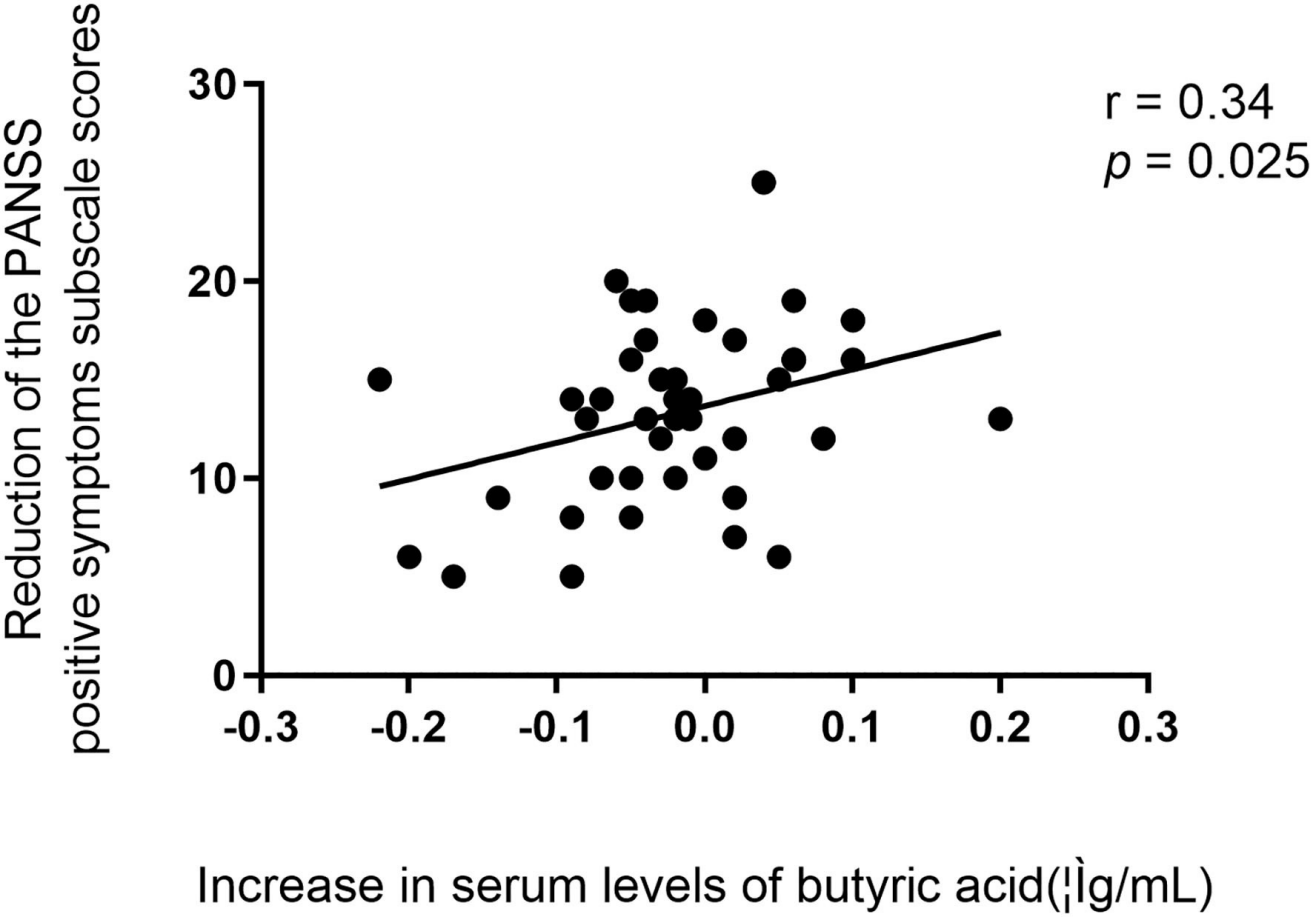
Relationship between baseline serum levels of butyric acid and the reduction ratio of the PANSS total scores and all subscale scores in patients.

We found that there were significant positive associations between baseline serum levels of butyric acid and the reduction ratio of the PANSS total scores (*r* = 0.44, *p* = 0.003), positive symptoms subscale scores (*r* = 0.36, *p* = 0.016), negative symptoms subscale scores (*r* = 0.35, *p* = 0.019), and general psychopathology subscale scores (*r* = 0.36, *p* = 0.016) (Figure 3). After controlling for confounding variables, all these correlations remained significant (*p's* < 0.05).

We further defined patients in two groups: good response (≥50% reduction ratio of the PANSS total score, *n* = 21) and poor response (<50% reduction ratio of the PANSS total score, *n* = 23). Stepwise logistic regression analysis indicated that higher baseline serum levels of butyric acid predict a better treatment response after controlling for baseline age, sex, education, duration of illness (odds ratio = 1.20, 95 % Confidence Interval 1.03–1.40, *p* = 0.017).

## Discussion

To our best knowledge, this is the first time to explore the serum levels of butyric acid in drug-naïve, first episode schizophrenia patients. The main findings of our study were that: (1) schizophrenia patients had increased serum levels of butyric acid after 24-week treatment; (2) the baseline serum levels of butyric acid were positively correlated with the reduction ratio of PANSS total and all subscale scores in schizophrenia patients; and (3) the increase in serum levels of butyric acid was positively correlated with the reduction of PANSS positive symptoms subscale scores after 24-week risperidone treatment in schizophrenia patients.

Previous studies indicated that ASD and schizophrenia may share some common biological basis and genetic vulnerability (23). Several studies have showed elevated fecal levels of butyrate in children with ASD (24, 25). In addition, animal studies have shown that butyrate is related to behavioral abnormalities, gastrointestinal discomfort, and conditioned aversions in the adult rodent model of ASD (26, 27). Taken together, clinical and preclinical researches showed abnormal levels of butyric acid in ASD. We failed to find abnormal levels of butyric acid in drug-naïve, first episode schizophrenia patients likely due to the heterogeneity of schizophrenia and the small sample size.

Recent studies have shown that butyrate may help normalize the behavioral and physiological abnormalities in ASD (28, 29). Schroeder et al reported that combined treatment with sodium butyrate and fluoxetine was superior to fluoxetine alone to achieve antidepressant-like effect (30). In addition, butyrate is helpful in alleviate cognitive deficits in premotor stage Parkinson's disease (31). The beneficial role of butyric acid in schizophrenia as suggested by our study might be related to its neuroprotective function. Butyrate plays an essential role in regulating synaptic plasticity and cortical development (32). There is growing evidence that links decreased synaptic consolidation and pruning in early childhood with both ASD and schizophrenia (33, 34). Since butyrate has been shown to reinvigorate synaptic plasticity in rodents, there is a strong interest in utilizing butyrate to treat a variety of neuropsychiatric conditions including schizophrenia (35). Mitochondrial dysfunction is considered to be involved in the pathophysiology of schizophrenia (4). Butyrate is capable of increasing mitochondrial activity, which can help to rectify the disease-associated mitochondrial dysfunction in the brain (36).

It has been well-established that dopamine is associated with positive symptoms of schizophrenia (37, 38). Some studies have suggested that butyrate modulates dopamine activity by inducing post-translational modifications of pre-existing CREB molecules in a cAMP/PKA-dependent manner to alter tyrosine hydroxylase transcription (39, 40). Another study showed that butyrate can alter the expression of catecholaminergic genes possibly through histone deacetylation or changes in mRNA stability (41). Moreover, studies have suggested that activation of the inflammatory response in both the peripheral blood and the central nervous system may play a key role in the pathogenesis of schizophrenia (42–44). It is known that elevated butyrate activity may contribute to the immune response (45). Butyrate can counteract LPS induced inflammation in primary microglia, hippocampal neurons, and neurons co-cultured with microglial or astrocytes in rats (46). In addition, butyrate may affect the balance between anti-inflammatory Treg and pro-inflammatory T helper 17 (Th17) cells (47). Treatment with butyrate can induce anti-inflammatory effect through G protein receptor 109a activation and downregulation of NF-κB activation (48), and therefore might be beneficial in individuals with schizophrenia (49).

The strengths of the present study include the use of a group of drug-naïve, first-episode schizophrenia patients with normal body weight and risperidone monotherapy. The selection of study participants in our study minimized potential confounding factors such as previous exposure to antipsychotic medications, variations in disease state (acute vs. chronic) or duration, differences in obesity and other metabolic disturbances at baseline. In longitudinal analyses, we take both non-completers and completers into account and a linear mixed model was used to calculated them. Several limitations of the present study should be noted. First, this study included a relatively small sample size and a short time duration of treatment. Furthermore, some important factors related to SCFAs, such as food intake and intestinal microbial condition, were not measured.

In summary, our study suggests that increased serum levels of butyric acid might be associated with a favorable treatment response in drug-naïve, first episode schizophrenia. Therefore, approaches that affect butyric acid metabolism, for example, a change in gut microbiota, may be considered as an adjunctive treatment for schizophrenia. Future studies further examining the mechanism by which butyric acid may affect the development and treatment of schizophrenia are warranted.

## Data Availability Statement

The original contributions presented in the study are included in the article/supplementary files, further inquiries can be directed to the corresponding author/s.

## Ethics Statement

The studies involving human participants were reviewed and approved by the Human Ethics Committee of the First Affiliated Hospital of Zhengzhou University, China (Approval No. 2016-LW-17). The patients/participants provided their written informed consent to participate in this study.

## Author Contributions

XL and XF designed the study and wrote the manuscript. XL, XY, and LP were in charge of conducting clinical assessment, collecting fasting blood samples, and doing experiments. XL and SH undertook the statistical analysis and wrote the first draft of the manuscript. YW, XH, and XS were responsible for supervision and reviewing. All authors contributed to and have approved the final manuscript.

## Conflict of Interest

The authors declare that the research was conducted in the absence of any commercial or financial relationships that could be construed as a potential conflict of interest.

## Publisher's Note

All claims expressed in this article are solely those of the authors and do not necessarily represent those of their affiliated organizations, or those of the publisher, the editors and the reviewers. Any product that may be evaluated in this article, or claim that may be made by its manufacturer, is not guaranteed or endorsed by the publisher.
